# Molecular screening for Epstein Barr virus (EBV) among Sudanese patients with nasopharyngeal carcinoma (NPC)

**DOI:** 10.1186/s13027-015-0002-4

**Published:** 2015-02-17

**Authors:** Hussain Gadelkarim Ahmed, Rania Saad Abdul Gader Suliman, Mohammed Siddig Abd El Aziz, Fawaz D Alshammari

**Affiliations:** Department of Pathology, College of Medicine, Molecular Diagnostics and Personalized Therapeutics Unit, University of Hail, Hail, Kingdom of Saudi Arabia; Department of Histopathology and Cytology, FMLS, University of Khartoum, Hail, Sudan; Department of Histopathology and Cytology, Faculty of Medical Laboratory Science, Sudan University for Science and Technology, Khartoum, Sudan; Department of Medical Laboratory Science, College of Applied Medical Science, University of Hail, Hail, Kingdom of Saudi Arabia

**Keywords:** Epstein barr virus, Nasopharyngeal carcinoma, Sudan

## Abstract

**Objective:**

The aim of this study was to screen for the presence of Epstein Barr Virus (EBV) among Sudanese patients with Nasopharyngeal Carcinoma (NPC).

**Methods:**

In this study, 150 tissue samples that were previously diagnosed as having NPC were screened for the presence of EBV using Polymerase Chain Reaction (PCR). PCR was performed to amplify two viral genes; EBV nuclear antigen-4 (EBNA-4) and latent membrane protein-1 (LMP1).

**Results:**

EBV genes were detected in 92/150 (61.3%) tissue samples. Of the 92 infected samples, 58/92 (63%) were found among males and 34/92 (37%) were among females.

**Conclusion:**

EBV is prevalent in the Sudan and responsible of the vast majority of cases of NPC.

## Introduction

NPC is the most common cancer arising from the nasopharynx that varies significantly from other cancers of the head and neck in its occurrence, causes, clinical behavior, and treatment [1].

NPC is infrequent in the United States and many other countries, representing less than 1 case per 100,000 in most populations, but is exceptionally common in southern regions of China [2], mainly in Guangdong, accounting for 18% of all cancers in China [3]. The etiology of NPC is multifactorial with race, genetics, environment and EBV as a major risk factor. While rare in Caucasian populations, it is one of the most common nasopharyngeal cancers in Chinese, and has endemic clusters in Alaskan Eskimos, Indians, and Aleuts. Remarkably, as native-born Chinese migrate, the incidence diminishes in successive generations, although still higher than the native population [4].

NPC caused by an interaction between infection with EBV and environmental and genetic factors, encompassing a multistep oncogenic process [5]. EBV has worldwide dissemination, infecting over 95% of the adult population worldwide [6]. In some parts of Asia, 80% of children are infected by 6 years of age, and almost 100% have seroconverted by 10 years of age [7]. Although primary EBV infection is characteristically sub-clinical, the virus is linked to the later progress of numerous malignancies, including NPC [3]. The virus is transmitted by saliva, and its primary infection occurs during childhood with replication of the virus in the oropharyngeal lining epithelial cells, followed by a latent infection of B lymphocytes (primary target of EBV). High titers of EBV-related antigens (specifically of IgA class), a latent EBV infection recognized in neoplastic cells of almost all cases of NPC. Moreover, the clonal EBV genome constantly identified in invasive carcinomas and high-grade dysplastic lesions propose a critical role of EBV in the pathogenesis of NPC in endemic areas [7,8]. Therefore, the aim of this study was to screen for EBV among Sudanese patients with NPC.

For identification of EBV we demonstrated **Epstein–Barr nuclear antigen 1** (EBNA1) and Epstein–Barr virus latent membrane protein 1 (LMP1) EBV genes. EBNA1 is a multifunctional, dimeric viral protein associated with EBV [9]. It is the only EBV protein found in all EBV-related malignancies [10]. LMP1 is the best-documented oncoprotein of the EBV latent gene products, as it is expressed in most EBV-related human cancers [11].

## Methods

In this study, 150 formalin fixed paraffin wax processed tissue samples of nasopharyngeal carcinoma were obtained from previously operated patients from different histopathology laboratories in Khartoum State, Sudan. All tissue samples were from those who had not yet given anti-cancer therapy. The study was approved by the Ethical Committee of the Research Board of Faculty of Medical Laboratory Science, Sudan University for Science and Technology, Khartoum, Sudan.

### DNA extraction

DNA was extracted from paraffin-embedded samples, by immersing tissue section in xylene to dissolve the paraffin from the tissue, and then rehydrated using a series of ethanol washes. Proteins and harmful enzymes such as nucleases were digested by proteinase K. Buffer containing denaturing agent (sodium dodecyl sulfate (SDS)), was added to facilitate digestion [12]. Nucleic acids were purified from the tissue lysate using buffer-saturated phenol and high speed centrifugation. Following phenol extractions, RNase A was added to eliminate contaminating RNA. Additional phenol extractions following incubation with RNase A were used to remove any remaining enzyme. Sodium acetate and isopropanol were added to precipitate DNA, and high speed centrifugation was used to pellet the DNA and facilitate isopropanol removal. Washing with 70% ethanol was performed to remove excess salts, followed by centrifugation to re-pellet the DNA [13,14]. DNA is re-suspended in distilled water, quantified and stored at −20°C Purified DNA was subsequently used in downstream applications of PCR.

### DNA quantification

To evaluate the DNA quantification after DNA extraction, we had analyzed DNA measurement using a NanoDrop spectrophotometer.

### PCR

EBV genome was detected by PCR using two primers that targets EBV nuclear antigen-4 (EBNA-4) 5′-GAGGAGGAAGACAAGAGTGG and 5′GATTCAGGCGTGGTCCTTGG 3′ and latent membrane protein-1 (LMP-1) 5′CCGAAGAGGTTGAAAACAAA3′and 5′GTGGGGGTCGTCATCATCTC 3′.

Any case detected EBNA-4 and/or LMP-1was considered positive indicating the presence of EBV.

## Molecular identification by polymerase chain reaction

Polymerase chain reaction (PCR) was carried out for amplification of target EBV genome by using genomic DNA template (1.5 L of EBV detection). PCR was performed in a total volume of 23 L for EBV. Master mix that contained 1 L of 10 mM dNTP mixed (2.5 mM dATP, 2.5 mM dGTP, 2.5 mM dCTP, and 2.5 mM dTTP), 1.5 L of 25 mM MgCl_2_, 2.5 L of 10 PCR buffer (10 m MTrisHCl [pH 8.3], 50 mM KCl), 1 L Taq polymerase (approximately 1 U), 1.5 L forward primer and 1.5 L reverse primer. Volume was completed to 25 L per reaction mixed with doubled distilled water (ddH_2_O). The master mix of all samples was mixed by vortexing in a sterile 0.2-mL PCR tube. Amplification was then set in an initial denaturation stage at 94°C for 5 minutes, then 30 cycles of 94°C for 30 seconds, and annealing at 55°C for 30 seconds, followed by ex- tension at 72°C for 90 seconds and a final extension stage at 72°C for 5 minutes.

### Ethical consent

The study was approved by Faculty Research Board, Faculty of Medical Laboratory Science, Sudan University for Science and Technology. This in addition to the fact that, the authors followed the tenants of the Declaration of Helsinki.

## Results

**Figure 1:**
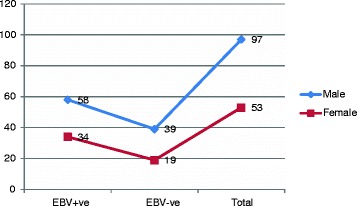
**Description of the study population by sex and EBV test (positive + ve or negative –ve).**

In this study we investigated 150 formalin fixed paraffin wax tissue blocks obtained from patients previously diagnosed with nasopharyngeal carcinoma, their ages ranging from 17 to 88 years with a mean age of 51 years. Male/female ratio was 1:83 to 1.00. Of the 150 NPC tissue specimens, EBV was identified in 92/150 (61.3%) samples and couldn’t be identified in 58/150(38.7%) tissue samples. Out of the 92 infected samples, 58/97 (60%) were found among males and 34/53(64%) were found among females. The 95% confidence interval and the odd ratio for sex male/female was 0.831(0.416-1.661), P < 0.365, as indicated in Figure 1.

**Figure 2:**
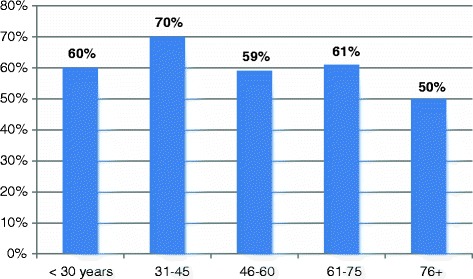
**Description of percentage of EBV positive test in each age group.**

As shown in Table 1, the highest frequency of infection rates were seen among age group 31–45 years representing 23/92 (25%) followed by age range 46–60 and 61–75 years, constituting 22/92 (23.9%) for each, then <30 years and 71+ representing 18/92 (19.5%) and 7/92 (7.6%), in this order. Nevertheless, when calculating the percentage in each group, the greatest proportion of infection was found in age group 31–45 years constituting 70% followed by 61–75, 31–45, <30 and 76+ years, representing 61%, 60%, 59% and 50%, respectively, as indicated in Figure 2.

**Table 1:** **Distribution of the study population by age and EBV infection**

**EBV**	**<30 years**	**31-45**	**46-60**	**61-75**	**76+**	**Total**
**+ve**	18	23	22	22	7	92
**-ve**	12	10	15	14	7	58
**Total**	30	33	37	36	14	150

**Figure 3:**
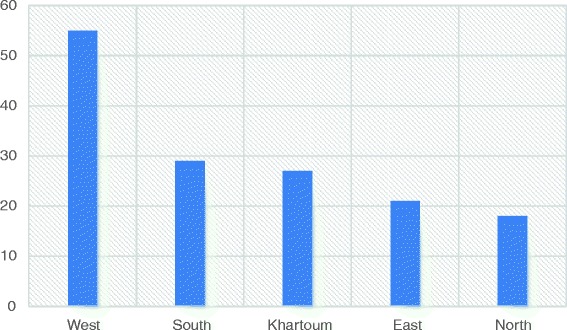
**Description of residence by NPC.**

**Figure 4:**
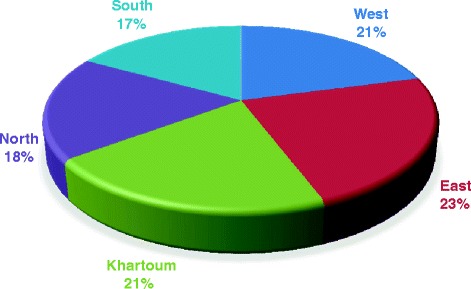
**Description of study population by residence and EBV infection.**

In respect to the residence, most of cases of NPC were coming from Western Sudan representing 55/150 (36.7%) followed by Southern Sudan constituting 29/150 (19.3%), as shown in Figure 3. In regard to the residence and EBV infection, the great majority of infections were identified among Western populations, representing 35/92 (38%) followed by Khartoum, (Eastern and Sothern), and Northern constituting 17/92 (18.5%), 15/92 (16.3%), and 10/92 (10.8%), respectively, as shown in Table 2. However, when calculating the percentage within individual entire residence, the utmost proportion of infection was found in the East representing 71.4% followed by West, Khartoum, North and South constituting 63.6%, 63%, 55.6% and 51.7%, in this order, as indicated in Figure 4.

**Table 2:** **Distribution of the study population by residence and EBV infection**

**Residence**	**EBV**	**Negative**	**Total**
	**positive**		
West	35	20	55
Khartoum	17	10	27
East	15	6	21
South	15	14	29
North	10	8	18
Total	92	58	150

## Discussion

As in many malignant tumors, NPC development reflects the multifarious interaction between host genes and environmental factors, but the crucial role of EBV infection delivers key vision into the etiology of this tumor. In this study, the correlation between NPC and the existence of the EBV genome was examined. An extremely significant positive correlation was found based on two genes encoding for EBV viral proteins. A number of studies have studied this correlation, but findings are greatly contradictory. The bulk of these studies reported a wide range of frequencies for the presence of EBV in NPC [15-17]. Many studies have investigated the association between EBV infection and nasopharyngeal carcinogenesis development [18]. The prevalence of histology types are different comparing endemic and non-endemic regions. In endemic areas, Type III represents over 97% of the cases, while the keratinizing type is more common in western countries (~75%) [19,20].

However, the role of EBV infection in the etiology of NPC in Sudan had been reported since 1979 [21], but none of these old studies have used molecular identification of the virus. A recent a study has investigated 43 biopsies obtained from Sudanese patients with NPC for the presence of EBV using EBER-ISH. Ten normal samples were used to assess the presence of the virus in non-cancer tissues. All nasopharyngeal carcinoma biopsies (100%) were positive for EBER1 in almost all carcinoma cells. No hybridization was observed in all 10 non-cancer tissues [22]. These findings greatly differ from our obtained prevalence of EBV in the present study (61.4%) and this might be attributed to their small sample size 43 compared to our sample size 150 tissue samples. Inconsistencies in detection efficacy may also be due to technical variances or variation between NPC subgroups. Usually, PCR is valuable in the detection of EBV in a certain cancer, but it might not be suitable for identifying the association between the virus and the particular type of cancer. However, Adam et al. applied EBV-encoded RNA (EBER) in Situ hybridization (EBER-ISH). The EBER-ISH is a novel method for precise detection of the EBV genome in the nuclei of tumor cells [23]. EBER-ISH has reported to have a sensitivity of 98% and a specificity of 100% in detecting primary NPC [16]. In the present study, all tissue samples were including not only tumor cells but also tumor infiltrating lymphocytes were investigated in PCR. However, EBV DNA may occasionally be detected in non-neoplastic nasopharyngeal tissues, particularly in ISH-negative cases probably reflect a lower sensitivity than PCR, particularly when a small number of viral copies are present [24]. Notably, a study applied ISH method, no virus was detected in the normal lymphocyte and epithelium surrounding the tumor [16].

Nevertheless, there are other studies reporting conflicting findings in other cancer types. Of these studies EBV was detected in 118/175 (67.4%) oral brush samples from apparently healthy volunteers and 69/217 (31.8%) of patients with oral cancer [25]. EBV LMP1 gene transcripts were found in 29 (36.3%) of the 80 patients with leukemia but in none of the healthy controls (P < .0001) [26]. EBV genome was detected in 55.5% (n = 90) of breast cancer tissues as compared to 23% in control tissue samples (p = 0.0001) [27].

Regarding sex, the number of males with NPC was higher than females, and this has been previously reported from Sudan [28,29]. Moreover, in this study, though the frequency of EBV infection was higher among males but when comparing the percentage of EBV infection within each group, women (64%) were relatively infected more than men (60%). Several studies have shown that NPC is more often diagnosed in men than in women, and tends to occur at an earlier age than do most cancers [30-32].

In this study, the infection was detected in relatively high numbers of patients at earlier adults’ age range. On the other hand most studies investigated relationship between EBV and gender; they found the proportion of infection is greater among men than women [33]. A previous study from Sudan have reported that NPC tended to occur in younger patients (youngest, 3 years), with 14 and 12.1% of cases in children 14 years or under in the Sudan Cancer Register and Radiation and Isotope Centre, Khartoum, respectively; it is the commonest childhood malignancy in the Sudan [28]. The association between age and presence NPC and EBV infection might be attributed to the early exposure to the virus which may somehow be linked to Burkitt’s lymphoma and its link to factors enhancing EBV infection. We mean the intense growing of Milk Bush (Euphorbia) particularly in the western parts of Sudan, which accelerate chance of infection with EBV.

According to the residence, most of cases of NPC in the present study were from Western Sudan, although Khartoum hosts the bulk of population with wide spectrum of ethnic diversity. An earlier study has reported high frequencies of NPC from Western Sudan, particularly, areas surrounding Nuba Mountains in Kordofan State [29]. However, they attributed that to the high background radiation due to naturally produced radioactive uranium rather than EBV infection. Geological studies revealed that radiation exposure rates around the Nuba Mountains are among the highest in the world in regard to background radiation. Radiation exposure in the Nuba Mountains is chiefly due to uranium, a byproduct of usable rock phosphate fertilizers [34,35]. Nevertheless, when comparing the proportions of EBV infection within each geographical residence, the highest percentage was observed among those coming from Eastern Sudan. A previous study from Sudan, studied the epidemiology and etiology of NPC has revealed that, the ethnic and geographical distribution of the cases showed that racial susceptibility played a significant role in the etiology of NPC in the Sudan with EBV as an exciting factor [28].

Nevertheless, and due to paucity of data and registry, the only epidemiological available report return to 1983 in which, 374 cases of NPC were recorded in the Sudan Cancer Registry (SCR) and 512 cases were seen at the Radiation and Isotope Centre, Khartoum (RICK)d. NPC formed 5.8% of all cancer cases in the SCR and 7.2% at the RICK; this is the highest frequency so far reported outside the Chinese at that time [28]. However, the current prevalence is expected to be high, particularly with increasing cases of Burkitt lymphoma in Sudan, though the reports in this context are very old. During the period 1962–73 twenty-nine cases of Burkitt’s lymphoma were seen and examined histologically in Khartoum, Sudan. Burkitt’s lymphoma formed 20% of the cases of childhood lymphomas [36].

In conclusion, the present study providing strong evidence supporting the etiologic role for EBV infection in NPC in Sudan. The relationship between age at onset of NPC, ethnic factors, geographical distribution and EBV infection requires further investigations. The outcomes of this study can be further utilized in terms of the new approaches in EBV vaccination and therapeutic consequences.
